# Data Cleansing and Sub‐Unit‐Based Molecular Description Enable Accurate Prediction of The Energy Levels of Non‐Fullerene Acceptors Used in Organic Solar Cells

**DOI:** 10.1002/advs.202308652

**Published:** 2024-02-22

**Authors:** Ting Zhang, Joshua Yuk Lin Lai, Mingzhe Shi, Qing Li, Chen Zhang, He Yan

**Affiliations:** ^1^ Department of Computing The Hong Kong Polytechnic University 11 Yuk Choi Road, Hung Hom, KLN Hong Kong 999077 China; ^2^ Department of Chemistry Hong Kong University of Science and Technology Clear Water Bay, Kowloon Hong Kong 999077 China

**Keywords:** data cleansing, non‐fullerene acceptors, organic solar cells, prediction of energy levels

## Abstract

Non‐fullerene acceptors (NFAs) have recently emerged as pivotal materials for enhancing the efficiency of organic solar cells (OSCs). To further advance OSC efficiency, precise control over the energy levels of NFAs is imperative, necessitating the development of a robust computational method for accurate energy level predictions. Unfortunately, conventional computational techniques often yield relatively large errors, typically ranging from 0.2 to 0.5 electronvolts (eV), when predicting energy levels. In this study, the authors present a novel method that not only expedites energy level predictions but also significantly improves accuracy , reducing the error margin to 0.06 eV. The method comprises two essential components. The first component involves data cleansing, which systematically eliminates problematic experimental data and thereby minimizes input data errors. The second component introduces a molecular description method based on the electronic properties of the sub‐units comprising NFAs. The approach simplifies the intricacies of molecular computation and demonstrates markedly enhanced prediction performance compared to the conventional density functional theory (DFT) method. Our methodology will expedite research in the field of NFAs, serving as a catalyst for the development of similar computational approaches to address challenges in other areas of material science and molecular research.

## Graphical abstract

An accurate and rapid computational method is developed to predict the energy levels of nonfullerene acceptors, yielding smaller prediction errors than previous computation methods. The accurate prediction is enabled by the combination of a data‐cleansing protocol that can effectively eliminate problematic experimental and a sub‐unit‐based molecular description method that greatly simplifies the complexity of molecular representations and hence the relevant molecular computation.

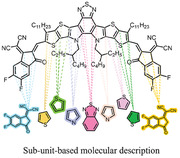

## 1 Introduction

In recent years, significant research progress has been made in the field of organic solar cells (OSCs) enabled by high‐performance non‐fullerene acceptors (NFAs), represented by the famous NFA molecule name Y6.^[^ ^1^ ^]^ Now, nearly all high‐efficiency (>18%) OSCs are based on Y6‐type NFAs.^[^ ^2^, ^3^, ^4^, ^5^, ^6^, ^7^, ^8^, ^9^ ^]^ Although Y6‐type NFAs have achieved dominant success, research on them still largely relies on trial‐and‐error methods, and their development has been relatively slow in comparison to competitive technologies like perovskite solar cells.^[^ ^10^ ^]^ There is currently a lack of dependable computational methods within our research community that can predict material properties and accelerate the advancement of NFAs.

Out of various material properties, predicting the energy levels (such as HOMO and LUMO) of NFAs is especially important, as even a minor alteration in HOMO/LUMO levels can result in significant changes in device efficiencies.^[^ ^11^, ^12^, ^13^, ^14^ ^]^ For instance, the HOMO energy offsets in state‐of‐the‐art PM6:Y6 systems are generally in the range of 0.1–0.15 eV.^[^ ^15^, ^16^, ^17^, ^18^, ^19^, ^20^, ^21^, ^22^ ^]^ Achieving OSC efficiencies beyond 20% requires precise adjustment and minimization of the HOMO offset between the donor and acceptor molecules. This can be achieved by making slight upward adjustments of the NFA's HOMO in increments of 0.05 eV or smaller. Therefore, the ability to accurately predict and manipulate the HOMO–LUMO levels of NFAs is a crucial aspect in the quest for higher OSC efficiencies.

In the field of organic solar cells (OSCs), the widely adopted computational method is density functional theory (DFT) calculation.^[^ ^23^, ^24^, ^25^ ^]^ However, it has been observed that DFT calculations often introduce significant errors when predicting the energy levels, specifically the highest occupied molecular orbital (HOMO) and lowest unoccupied molecular orbital (LUMO), of non‐fullerene acceptors (NFAs).^[^ ^26^, ^27^ ^]^ For instance, in several influential publications from reputable research groups, the HOMO/LUMO levels obtained through DFT calculations have deviated from the experimental HOMO/LUMO data by as much as 0.14 and 0.56 electronvolts (eV) respectively (as summarized in Table S1, Supporting Information).^[^ ^18^, ^28^, ^29^ ^]^ The commonly used experimental method in the OSC community is cyclic voltammetry (CV) which measures the redox potentials of a NFA molecule, which are then used to calculate HOMO/LUMO levels of NFAs. The deviations between HOMO/LUMO values by DFT calculations and CV measurements are in part because DFT calculation is computing the energetic state of isolated molecules in the gas phase, while CV measures the properties of the pi‐pi stacked molecules in the solid state.^[^ ^30^ ^]^ (Note that the prediction errors of DFT calculation on LUMO levels are particularly large, yet the LUMO level of the acceptor is important in influencing the Voc of the OSC devices). Alternatively, various machine learning (ML) methodologies have been used to predict the properties of materials and efficiencies of OSCs,^[^ ^31^, ^32^ ^]^ which have been summarized in previous review articles. Note that many past reports were focused on donor molecules used in the previous generations of fullerene‐based OSCs,^[^ ^33^, ^34^ ^]^ and there are relatively fewer reports on property prediction for state‐of‐the‐art NFA molecules. Among recent prediction work on NFAs, Sun and coworkers developed a strategy by cutting NFA molecules into several segments and using ML methods to predict the OSC efficiencies (not energy levels).^[^ ^35^ ^]^ To the best of our knowledge, there are yet successful models reported that can predict the HOMO/LUMO levels of NFAs accurately and rapidly.

Another challenge of accurate prediction is related to experimental data of HOMO/LUMO levels that are typically obtained through CV measurements, which have significant measurement errors. For instance, a comparison of CV measurement results from different groups for the same material, as presented in Table S2 (Supporting Information), shows variations in HOMO/LUMO values ranging from 0.18 to 0.21 eV.^[^ ^36^, ^37^ ^]^ To address the problem of large errors, it will be necessary to perform data cleansing and improve the quality of the CV data before inputting to ML models.

In this paper, we report a novel computational method that can predict the HOMO/LUMO levels of NFAs both accurately and rapidly. The first component of our methodology involves data cleansing which reduces the experimental errors of HOMO/LUMO measured by CV (labeled as HOMO_CV_/LUMO_CV_ in this paper). Although the HOMO_CV_/LUMO_CV_ data can have substantial errors, other associated experimental data, such as open circuit voltage (Voc) and bandgap, are much more accurate. As there is a well‐established correlation between HOMO/LUMO and Voc/bandgap, we can use Voc and bandgap data to indirectly estimate the HOMO/LUMO levels of NFAs (labeled as HOMO_Voc_/LUMO_Voc_). If the HOMO_CV_/LUMO_CV_ data are accurate, then there should be relatively small differences between HOMO_CV_/LUMO_CV_ and HOMO_Voc_/LUMO_Voc_. In the contrary, if there is a large difference between the HOMO_CV_/LUMO_CV_ and HOMO_Voc_/ LUMO_Voc_ for a specific NFA, there must be significant experimental errors in either the HOMO_CV_/LUMO_CV_ or Voc/bandgap data, with the former possibility being more likely. Therefore, we can use the discrepancy between HOMO_CV_/LUMO_CV_ and HOMO_Voc_/LUMO_Voc_ as an indicator to identify problematic data and improve data quality.

For the second component of our method, we develop a sub‐unit‐based molecular description method to describe NFAs and then use it as input to ML models. In specific, as NFAs are a specific type of molecules that contain fused or linearly linked aromatic units, we divide NFA molecules into fewer than ten aromatic sub‐units and use the electronic properties of the sub‐units as numeric description code as inputs for ML models. With our data cleansing procedures and sub‐unit‐based molecular description, we utilize several existing ML models to predict the HOMO/LUMO levels of NFAs. The best prediction result was achieved using the gradient boost regression trees (GBRT) model, with which we demonstrate fast (the computation can complete within several seconds using a common office laptop) and accurate (prediction error only ≈0.06 eV) predictions of the HOMO/LUMO levels of NFAs that are not achievable using conventional DFT computational method.

### 1.1 Our Methodology – Component #1 Data Cleansing

To improve the quality of experimental data by CV, we perform cross‐calibration of CV data with other experimental data that are more accurate. The majority of OSC research articles report optical bandgap (Eg) and Voc of solar cells. These are two device parameters that typically exhibit great accuracy and small discrepancies if we compare the results reported by different research groups. (Presented in Table S3 is a compilation of Voc and Eg data for the same PM6:Y6 material set, reported by different research groups. This summary demonstrates minimal variations between the different groups, with a standard deviation of only 0.010–0.015 eV for Voc and Eg as shown in Table S3, Supporting Information). It is thus clear that the Voc and Eg data have much smaller errors than the HOMO/LUMO data measured by CV. Then we can use the correlation between HOMO/LUMO and Voc/bandgap to estimate HOMO/LUMO based on the following equation:^[^ ^38^ ^]^

(1) $$\mathrm{Voc}=\mathrm{HOMO}\left(\mathrm{donor}\right)-\mathrm{LUMO}\left(\mathrm{acceptor}\right)-\mathrm{voltage}\mathrm{loss}$$

The HOMO values of conventional donor materials are well documented in the literature. Although HOMO values of donor materials could have significant variation between different research groups, we can take the average of reported values and assume certain standard HOMO values for every donor material reported in the literature. Consequently, the sole remaining variable in Equation (1) is voltage loss, typically ≈0.55–0.6 eV for most high‐efficiency OSCs. In this paper, we assume that the voltage loss is 0.6 eV. Utilizing Equation (1), the LUMO values of acceptors can be determined with a good degree of precision, which should fall within 0.1 eV. Next, the HOMO level of NFAs can be determined with minimal errors using the following equation, as the experimental error for Eg is very small.

(2) $$\mathrm{HOMO}\left(\mathrm{acceptor}\right)=\mathrm{Eg}-\mathrm{LUMO}\left(\mathrm{acceptor}\right)$$

To compile our dataset of HOMO/LUMO values, we collected data from two sources: values obtained through CV measurements in the original literature, which we label as HOMO_CV_ and LUMO_CV_, and values estimated using Equations (1) and (2) based on Voc and Eg (bandgap) calculations, which we refer to as HOMO_Voc_ and LUMO_Voc_ in this paper. To quantitatively assess the deviations between HOMO_CV_/LUMO_CV_ and HOMO_Voc_/LUMO_Voc_, we introduced two new parameters: ΔHOMO, calculated as HOMO_Voc_ – HOMO_CV_, and ΔLUMO, calculated as LUMO_Voc_ – LUMO_CV_. If the ΔHOMO or ΔLUMO of a certain NFA is larger than a certain threshold, we can disqualify the HOMO_CV_/LUMO_CV_ and HOMO_Voc_/LUMO_Voc_ and remove such problematic data from our database. Through the implementation of this quality assurance and elimination process, we eliminated ≈30% of the original 1000 NFA molecules and obtained a smaller set of more reliable data, which consisted of ≈600–700 NFAs.

### 1.2 Our Methodology‐ Component #2 Subunit‐Based Description

**Figure 1:**
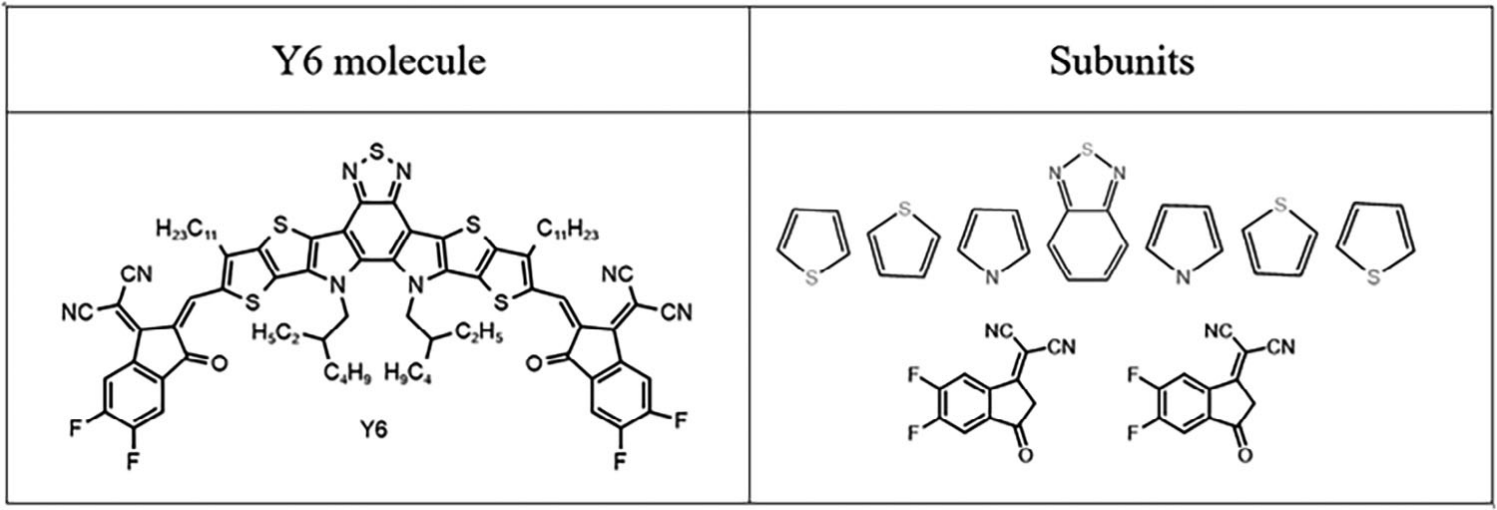
Molecular structure of the famous non‐fullerene acceptor named Y6. The structure of Y6 is divided into several subunits including thiophene, pyrrole, and benzothiadiazole units with two end groups.

NFA molecules can be generally described as a “linear combination of single or fused aromatic rings capped with two electron‐withdrawing end‐groups.” To simplify the molecular description of NFA molecules, we divide the chemical structures of NFAs into multiple sub‐units. For instance, the Y6 molecule, illustrated in **Figure** **1**, comprises seven fused aromatic rings (thiophene, pyrrole, and benzothiadiazole units) with two end groups. Then, we consider the following three aspects that can influence the HOMO/LUMO levels of the NFA molecules: electronic property of sub‐unit, form of linkage between subunits, and influence of side chains. The details of the three aspects are described as follows.

#### 1.2.1 Use Sub‐Unit Electronegativity (SEN) as Numeric Inputs

Computational chemistry has established that the energy levels of NFA molecules are primarily dependent on the sub‐units “electron negativity.” The term electronegativity was initially introduced to describe the electron‐donating or electron‐withdrawing effects of individual elements or atoms.^[^ ^39^ ^]^ Here we introduce a new concept of sub‐unit electron negativity (SEN), which represents the electron donating/withdrawing effects of the sub‐units, instead of individual atoms. In order to obtain appropriate initial values for the sub‐unit electron negativity (SEN), we leverage the DFT method. While DFT may not provide highly accurate predictions of the ultimate energy levels of the highly complicated NFA molecules, it can furnish reasonable estimates of the electronic properties of relatively simple sub‐units. As depicted in Table S4 (Supporting Information), we present the DFT‐calculated HOMO/LUMO values for the prevalent sub‐units utilized in NFAs, which will subsequently serve as inputs for the SEN values of the sub‐units.

#### 1.2.2 Numeric Inputs Related to the Number of Nonfused Linkages

The number of nonfused single‐bond linkages in the molecular backbones is another crucial factor that could influence the HOMO/LUMO values of NFAs. While widely the used Y6 and ITIC NFAs possess rigid molecular structures with zero rotatable nonfused linkages, a sub‐family of nonfused NFAs has emerged more recently, which features multiple rotatable single bonds.^[^ ^40^, ^41^, ^42^, ^43^, ^44^, ^45^ ^]^ Presented in Figure S1 (Supporting Information) is an illustrative instance of nonfused NFAs that contain freely rotatable single bonds. These single bonds, located in the molecular backbone and linking the four thiophene units in the central portion of the structure, significantly impact the HOMO/LUMO levels of NFAs when compared to the rigid and fully fused aromatic structure of Y6. According to our chemical knowledge, the presence of each nonfused single bond linkage will increase the HOMO/LUMO levels of the NFAs to some degree, which we estimate to be ≈0.1 eV. Hence, as an input, the number of nonfused linkages and this estimated influence value (0.1 eV) should be included, in addition to the SEN values of subunits.

#### 1.2.3 Numeric Inputs Related to Side Chains

The next aspect to consider is the impact of side chains or substitution groups on the HOMO/LUMO values of NFAs. It is well‐known from our chemical understanding that alkoxy side chains in specific positions can significantly affect the HOMO/LUMO values of NFAs, whereas alkyl side chains have a comparatively minor influence. Based on this knowledge, we estimate the possible effects of various side chains and include these values as inputs. Table S6 (Supporting Information) summarizes the different side chains and substitution groups and their estimated influences in a quantitative manner, which are also used as the inputs.

Presented here is an example of how to input an NFA structure into our ML models. The commonly studied NFA molecule Y6, depicted in Figure 1, is composed of nine sub‐units that are connected in a specific sequence, namely IC‐2F, thiophene, thiophene, pyrrole, benzothiadiazole, pyrrole, thiophene, thiophene, and IC‐2F. Following the calculation of the SEN values of all sub‐units using DFT, we obtain a series of SEN values that represent the Y6 structure (−3.4, −0.23, −0.23, −3.68, −2.35, −3.68, −0.23, −0.23, −6.35, −6.35, −6.78, −6.62, −6.78, −6.35, −6.35, −3.4). The simplicity of these inputs for our ML models indicates that the fitting of the model should be uncomplicated.

#### 1.2.4 Standardizing the Length of Numeric Inputs

Different NFAs can have different lengths of numeric inputs, which might increase the complexity of the computation. To standardize the length of the numeric inputs, we set the standard input length at 30 numeric values, which corresponds to 15 sub‐units, as each unit has two numeric values for inputs. If an NFA has less than 15 submits, then the input of this NFA would have less than 30 numeric values. We just fill in the empty slots with zero, so that all NFAs have the same length of input. At this point, there are no NFAs reported in the OSC community, to the best of our knowledge, with more than 12 subunits, so this standard length of 30 numeric values should cover all the possible NFAs to date. If there are possibilities to extend to more than 15 units, we can simply modify our model parameter to accommodate the new computation needs.

## 2 Model Fitting and Validation

In our research, we employed a suite of seven machine learning methodologies, namely gradient boost regression trees (GBRT), random forests (RF), extremely randomized forests (ERF), decision trees (DT), artificial neural networks (ANN), support vector machines with a polynomial kernel (SVM (poly)), and long short‐term memory (LSTM) for predicting the energy levels of OSCs. To safeguard the credibility and precision of our results, we adopted a K‐Fold cross‐validation technique as a mean to evaluate the predictive performance of these models. For the process of model training and evaluation, the entire dataset was subjected to random shuffling before being partitioned into two distinct segments – training and testing datasets. The split ratio was kept at nine to one, representative of the training‐to‐testing data, a ratio that is frequently used in the realm of machine learning. Then the prediction results of our model are evaluated by three commonly used parameters, namely, the mean absolute error (MAE), the root mean squared error (RMSE), and the Pearson correlation coefficient (r). The MAE value provides a measure of the discrepancies between the true and estimated values. The RMSE value serves as an indicator of the model's accuracy, whereas the Pearson correlation coefficient (r) is a widely adopted correlation metric.

Similar to some of the previous studies,^[^ ^33^ ^]^ we also further test the performance of our prediction model in a practical “use‐case” scenario. In specific, we collected 20 commonly used NFAs and formed a “use‐case” dataset to test the performance of our model versus conventional DFT calculations. These molecules were specifically selected as they are commercially available, denoted by their respective Chemical Abstracts Service (CAS) numbers, and have both experimentally determined and DFT‐computed HOMO/LUMO energy levels documented in peer‐reviewed literature. It's noteworthy to mention that the experimental values within the “use‐case” dataset were exclusively procured by CV, the most common technique within the domain of organic electronics due to its relative simplicity and ease of execution.

## 3 Result

**Figure 2:**
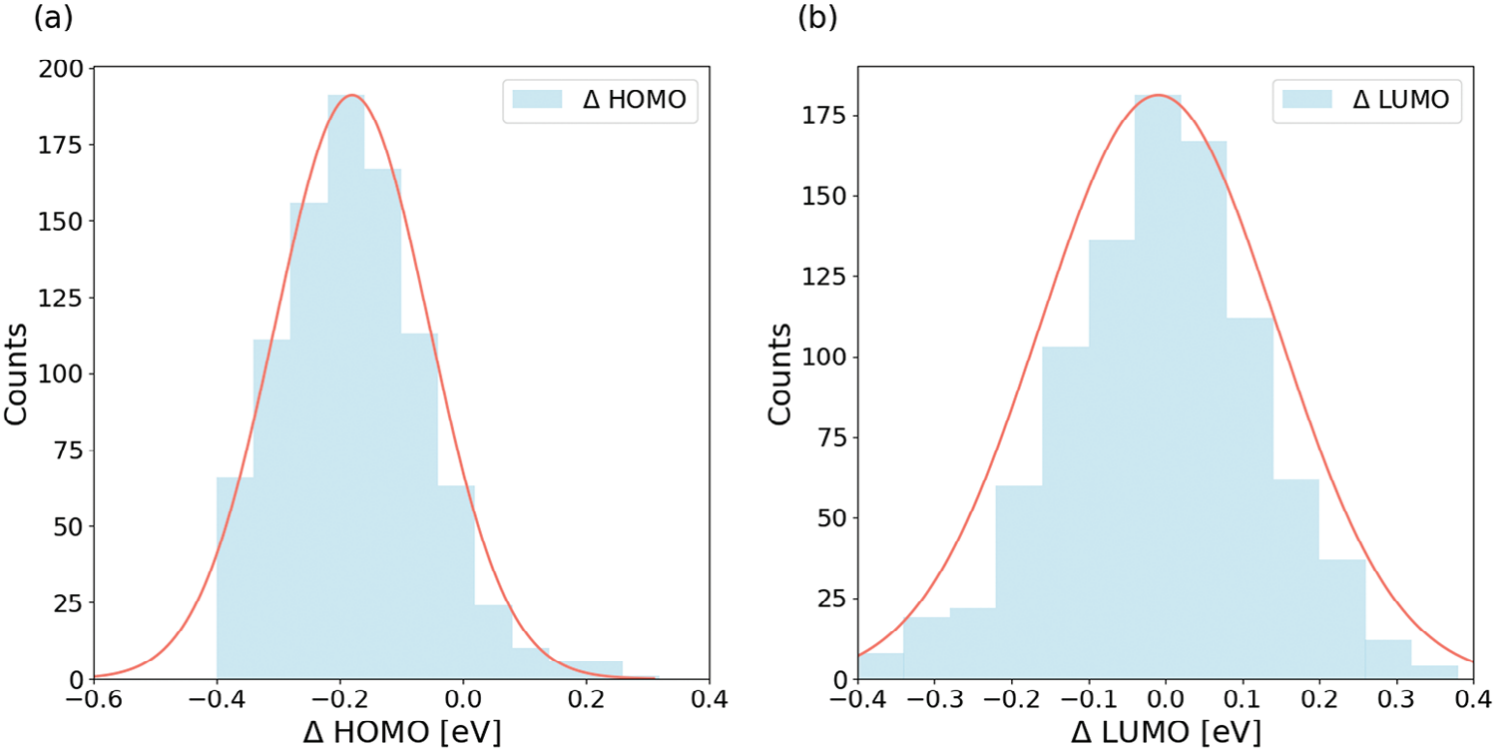
Illustration of the distribution of ΔHOMO and ΔLUMO. The definition of ΔHOMO and ΔLUMO are the differences of HOMO and LUMO values measured by cyclic voltammetry or estimated by Voc and Eg, with ΔHOMO = HOMO_CV_ – HOMO_Voc_ and ΔLUMO = LUMO_CV_ – LUMO_Voc_.

Among the seven aforementioned AI models, we show that the GBRT model yields the best prediction performance. Therefore, we focus on the GBRT model and use it on four different datasets without or with various data‐cleaning procedures. Here we first show the prediction results based on the GBRT model on the different datasets. Shown in **Table** **1** are the prediction performance using four different datasets in our study. While dataset #1 includes HOMO_CV_ and LUMO_CV_, dataset #2 includes HOMO_Voc_/LUMO_Voc_. To improve the quality of datasets #1 and #2, we use either ΔHOMO or ΔLUMO as the error indicator to identify problematic data in datasets #1 and #2. In **Figure** **2**a,b, we show the distribution histographs of ΔHOMO or ΔLUMO for ≈1000 NFAs, respectively. It can be seen that the distribution of ΔLUMO is slightly wider than that of ΔHOMO (standard deviations of ΔLUMO and ΔHOMO are 0.15 and 0.12 eV, respectively), which is consistent with the fact that LUMO measurement generally has larger experimental errors than HOMO. Also, the distribution of ΔLUMO is a better Gaussian distribution centered at the zero value of ΔLUMO, indicating that ΔLUMO is more influenced by random errors, while the distribution of ΔHOMO could be influenced by certain hidden systematic errors. For these reasons, we use ΔLUMO as the indicator and set the threshold at ≈1 standard deviation (0.15 eV) and eliminate ≈32% of data with ΔLUMO smaller than −0.15 eV or larger than 0.15 eV. Based on the ΔLUMO shown in Figure 2b, dataset #3 is formed after performing this data cleansing procedure on dataset #1, while dataset #4 is formed from dataset #2.

**Table 1:** Prediction performance of our methodology (based on the sub‐unit‐based molecular description and GBRT model) using four different datasets with gradually improved data quality. The parameters of prediction performance include MAE, RMSE, and r, which are widely used to indicate the accuracy and correlation of AI models.

Computation method	Datasets	MAE [eV]	RMSE [eV]	r
Subunit‐code description and GBRT	Data set #1, HOMO_CV_ LUMO_CV_	0.082	0.116	0.990
	Data set #2, LUMO_Voc_ HOMO = LUMO_Voc_ + Eg	0.069	0.100	0.991
	Data set #3, data set #1 minus 30% less accurate data	0.070	0.097	0.993
	Data set #4, data set #2 minus 30% less accurate data	0.060	0.083	0.994

Note that we also attempted to use ΔHOMO as the indicator to identify problematic data and the average prediction error (MAE) is slightly larger than those obtained using ΔLUMO, with the MAE increased from 0.06 to 0.064 eV. We believe this is because there are particularly large errors in the measurement of LUMO for NFA materials. As we summarized in the manuscript, the errors of CV measurement are typically 0.2 eV for HOMO, but 0.5 eV for LUMO. Therefore, using LUMO levels as the indicator could best identify the problematic experimental data.

**Figure 3:**
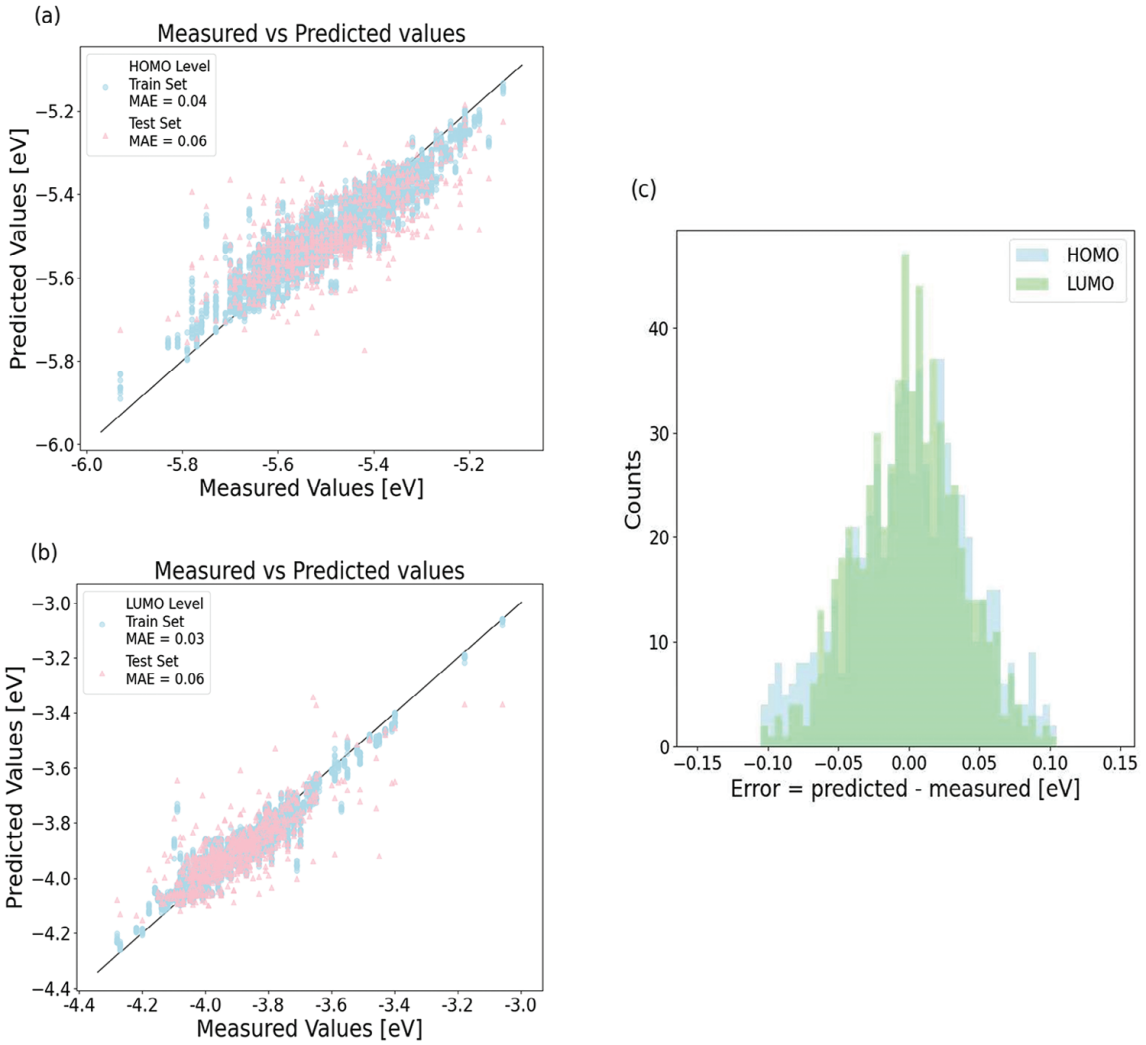
Results of AI model prediction using the optimized GBRT model and dataset #4. a,b), This shows the model predicted values versus the HOMO_Voc_ and LUMO_Voc_ data in dataset #4. The training set is shown as blue circles, while the test set is shown as red triangles. The insets of (a,b) show the MAE values of training and test sets. c), distribution of the prediction errors with the HOMO prediction error shown in green and LUMO in blue.

The prediction results in Table 1 include MAE, RMSE, and r, which are generally used to evaluate the performance of predictive models in a quantitative manner. These results clearly show two trends: 1) as datasets #3 and #4 outperform datasets #1 and #2, this means that our data cleansing procedure is indeed effective in reducing data error and improving the performance of prediction. In addition, the fact that datasets #2 and #4 yield better results than datasets #1 and #3 shows HOMO_Voc_/LUMO_Voc_ data can achieve better performance than those based on CV measurement (dataset #1). In addition, the detailed results of model training and predictions are shown in **Figure** **3**. It is clearly seen that the model training has very small MAE values of 0.03–0.04 eV for HOMO and LUMO and the MAE of the test sets is ≈0.06 eV, which is an impressive result compared to previous reports. As shown in Figure 3c, the prediction errors are mostly within −0.1 and 0.1 eV and the overall errors appear to exhibit a normal Gaussian distribution.

Next, we focus on the best dataset #4 explore various AI models, and compare the results obtained using SMILES or sub‐unit‐based molecular description. As shown in **Table** **2**, the GBRT model achieved the best predicting results, while the other models, especially the ANN models, are not suitable for the prediction of energy levels. The enhanced performance of GBRT and RF over ANN in certain scenarios can be linked to several key factors. First, GBRT and RF, being tree‐based

**Table 2:** Prediction performance of different AI models (GBRT, RF, ERF, DT, ANN, SVM, and LSTM) with either SMILES or our subunit‐based molecular descriptions as model inputs. The parameters of prediction performance include MAE, RMSE, and r.

model		MAE [eV]	RMSE [eV]	*r*
**Gradient boost regression tree (GBRT)**	Sub‐unit	**0.060**	**0.083**	**0.994**
	SMILES	0.102	0.140	0.980
Random forest (RF)	Sub‐unit	0.063	0.086	0.993
	SMILES	0.100	0.138	0.981
Extremely randomized forest (ERF)	Sub‐unit	0.064	0.091	0.993
	SMILES	0.099	0.138	0.981
Decision Tree (DT)	Sub‐unit	0.072	0.106	0.990
	SMILES	0.121	0.174	0.970
Artificial neural network (ANN)	Sub‐unit	0.111	0.157	0.978
	SMILES	0.730	0.979	0.586
Support vector machine (SVM)	Sub‐unit	0.089	0.112	0.991
	SMILES	0.109	0.143	0.989
Long Short‐Term Memory (LSTM)	Sub‐unit	0.098	0.121	‐
	SMILES	0.184	0.155	0.977

Algorithms are intrinsically more straightforward and easier to understand than the multifaceted, nonlinear nature of ANNs. This simplicity is especially advantageous when working with datasets characterized by a limited number of features and instances; under these conditions, GBRT and RF are less likely to overfit compared to ANNs. Furthermore, GBRT and RF can seamlessly accommodate both categorical and numerical features, whereas ANNs might necessitate more involved preprocessing for these data types. Finally, the training and fine‐tuning procedures for GBRT and RF are generally more time‐efficient and less complicated, standing in stark contrast to the extensive computational demands and intricate tuning processes associated with ANNs.

More importantly, the results in Table 2 show that our sub‐unit‐based molecule prediction shows significantly improved prediction results compared to the SMILES‐based description. As the performance of models using SMILES notations as inputs can be affected by factors such as sequence sensitivity and data preprocessing. Due to the nature of SMILES notations, where a single molecule can have multiple representations, the model may face challenges in learning and recognizing a wider range of patterns, thereby reducing its accuracy and efficiency. Additionally, during data preprocessing steps like tokenization, crucial chemical information could be lost, particularly when dealing with larger molecules that exceed the model's manageable sequence length, necessitating truncation or deletion of parts of the SMILES string. In some sense, our sub‐unit‐based molecular description is more suitable for the prediction of energy levels, as the electronic properties of the sub‐units are the pre‐dominant factors influencing the HOMO/LUMO levels of NFAs. Therefore, the sub‐unit‐based description can achieve fast and accurate prediction by avoiding over‐complication in computing the energy levels of large NFA molecules.

### 3.1 External Validation: Comparison with DFT Computation Using “use‐case” Dataset

Inspired by the “use‐case” validation used in previous work, we also further compare our model prediction performance against commonly used DFT computation using a “use‐case” dataset containing 20 commercially available NFA molecules that are considered “workhorse” materials for the OSC research community. The CV experimental data of these “use‐case” NFA molecules are available in the literature. The HOMO/LUMO levels of these molecules are both predicted using our method or the conventional DFT calculation (based on the commonly used hybrid functionals of B3LYP and 6–31G advanced basis sets). Both our model predictions and DFT calculation results are compared against the HOMO/LUMO values determined by the experimental CV method.

**Figure 4:**
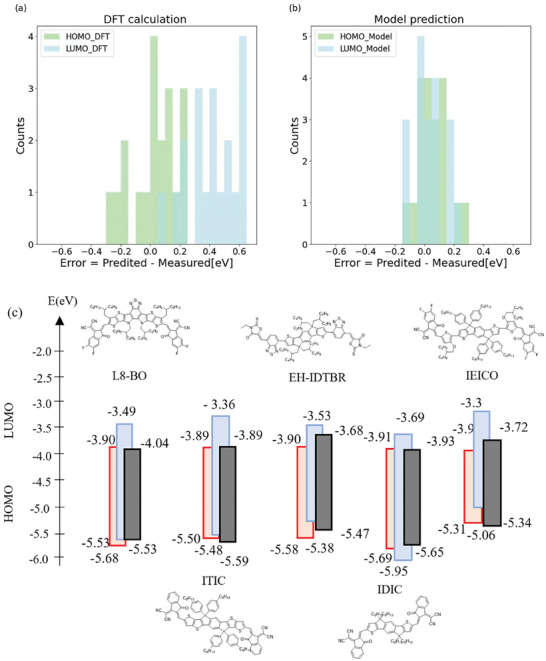
Results of testing our prediction method and model using “use‐case” dataset. a) the distribution of prediction errors by DFT calculations versus experimental data; b) the distribution of prediction errors by our optimized model/data set versus experimental data; c) Illustration of predicted HOMO/LUMO values by our model or DFT versus experimental data for several important non‐fullerene acceptors.

Shown in **Figure** **4** is the distribution of prediction errors by DFT and our model prediction. Our model clearly outperforms the DFT method significantly. In **Table** **3**, we summarize the prediction performance of our model against conventional DFT computation. Both MAE and RMSE values are significantly reduced using our model compared to those using DFT. In specific, our model appears to be particularly effective in reducing the predictive errors for LUMO. While the MAE/RMSE of LUMO prediction is 0.347/0.380 eV using DFT computation, our method enables dramatic improvement by reducing the MAE and RMSE to 0.064 and 0.085 eV, respectively. As discussed in the introduction session, conventional DFT computation suffers from large errors for LUMO predictions. As the LUMO level of the acceptor is an important parameter that can influence the Voc of OSC devices, our model will help us to predict the Voc of OSC devices. In Figure 4c, we show the HOMO/LUMO levels of five commonly used NFAs by CV measurement, DFT calculations, and our model prediction. Our model prediction results appear to agree with CV measurement reasonably well for these NFAs.

**Table 3:** Comparison of the performance of our predictive model versus conventional DFT computation for molecules in the selective “use‐case” testing dataset.

Method	MAE/RMSE of HOMO [eV]	MAE/RMSE of LUMO [eV]
This model	0.086/0.111	0.064/0.085
DFT	0.098/0.129	0.347/0.380

From these data, the prediction errors achieved using our method are significantly smaller than those achieved in previous computational or experimental methods. As mentioned in the introduction session, there have been previous studies to predict the energy levels of donor molecules in OSCs, but the prediction errors were typically ≈0.2 eV. These results further validate the success of our methods. As a next step, we also established an online APP (https://jokeviner.github.io/molecular/) that allows other researchers to use our prediction model. At this point, this APP is designed to predict the energy levels of NFAs only, as the available data are mostly on NFAs. In the future, we hope to expand the model and the online APP to a more diverse range of OSC materials such as donor polymers. We will distribute this online APP to the OSC researchers and hope to collect feedback that would likely help us further improve our prediction model.

In addition, those previous methods often rely on a large database such as the Harvard Clean Energy Project (CEP) database, which contains over 2 million molecules. Such a large database was needed because the training of the conventional AI models (using SMILES code as inputs) requires a large amount of data to achieve optimal data fitting. However, using our sub‐unit‐based molecular description as inputs, the model training in our study uses the data of only ≈700 NFA molecules. The reason why we can achieve accurate prediction using such a small database is probably due to the use of our sub‐unit‐based molecular description that simplifies molecular description and predicts the HOMO/LUMO levels more effectively. Our approach is particularly useful for NFAs, as the emergence of NFAs was quite recent and there are only ≈1000 NFAs available in the literature. In such a new research field, it is not possible to form a large database necessary for the training of conventional AI models. Therefore, our method becomes especially useful in situations where there are relatively small amount of data available.

Lastly, our method can also be used to predict Voc. After the prediction of HOMO and LUMO levels, the predicative values can be used to estimate the Voc of the solar cells based on Equation (1) shown in the previous page. Certainly, the Voc of organic solar cells will also be dependent on the choices and properties of donor polymers such as HOMO. In our database, we have the average HOMO values of most donor polymers, so these values can be used to estimate the Voc of organic solar cells. We show in Table S7 (Supporting Information) the predicted Voc values for the 20 NFAs in the “user‐case” validation. Compared with the experimental Voc values, the predicted Voc values have an average of deviation of ≈0.06 V, which is a respectable value comparable to the MAE of LUMO prediction. It is important to note that, in this Voc prediction, we assume that the voltage loss is 0.6 eV for all solar cell devices, which could cause some errors. If there could be a more precise prediction or estimation of voltage loss, then our Voc prediction accuracy can be further improved.

## 4 Conclusion

To summarize, we demonstrate fast and highly accurate computation of the HOMO/LUMO levels of NFAs used in OSCs. This accomplishment was made possible through novel data cleansing procedures and machine learning techniques. Taking a data‐centric approach, we first perform data cleansing on the experimental data from CV based on the correlation between problematic HOMO/LUMO data and more accurate Voc and bandgap data, which facilitated the elimination of unreliable HOMO/LUMO data and improved the data quality for machine learning model input. Next, we designed a subunit‐based molecular description, which substantially accelerated computation and improved prediction accuracy. We achieved fast computation, which could be completed in just 1 s using a common laptop computer, while simultaneously attaining the best prediction accuracy compared to any of the previous computational methods, despite only having data on 1000 NFAs molecules. As our method was successful with a smaller dataset, it has the potential to achieve even better prediction accuracy as more molecules are developed by the OSC research community in the future. The success of our method has the potential to inspire the research community to develop similar computational models to predict other important molecular properties, such as solubility, charge mobility, and device efficiency of OSCs.

## Conflict of Interest

The authors declare no conflict of interest.

## Author Contributions

T.Z. conceived of the presented idea, developed the theory and performed the computations. C.Z. and M.S. verified the analytical methods. H.Y. and J.L. investigate the data cleansing of this work. All authors discussed the results and contributed to the final manuscript.

## Supporting information

Supporting Information

## Data Availability

The data that support the findings of this study are available in the supplementary material of this article.

## References

[1] J. Yuan , Y. Zhang , L. Zhou , G. Zhang , H.‐L. Yip , T.‐K. Lau , X. Lu , C. Zhu , H. Peng , P. A. Johnson , M. Leclerc , Y. Cao , J. Ulanski , Y. Li , Y. Zou , Joule 2019, 3, 1140.

[2] W. Gao , F. Qi , Z. Peng , F. R. Lin , K. Jiang , C. Zhong , W. Kaminsky , Z. Guan , C.‐S. Lee , T. J. Marks , H. Ade , A. K.‐Y. Jen , Adv. Mater. 2022, 34, 2202089. 10.1002/adma.202202089 35724397

[3] Y. Wei , Z. Chen , G. Lu , N. Yu , C. Li , J. Gao , X. Gu , X. Hao , G. Lu , Z. Tang , J. Zhang , Z. Wei , X. Zhang , H. Huang , Adv. Mater. 2022, 34, 2204718. 10.1002/adma.202204718 35747988

[4] J. Yuan , T. Huang , P. Cheng , Y. Zou , H. Zhang , J. L. Yang , S.‐Y. Chang , Z. Zhang , W. Huang , R. Wang , D. Meng , F. Gao , Y. Yang , Nat. Commun. 2019, 10, 570. 30718494 10.1038/s41467-019-08386-9 PMC6362024

[5] G. Zhang , F. R. Lin , F. Qi , T. Heumüller , A. Distler , H.‐J. Egelhaaf , N. Li , P. C. Y. Chow , C. J. Brabec , A. K. Y. Jen , H.‐L. Yip , Chem. Rev. 2022, 122, 14180. 35929847 10.1021/acs.chemrev.1c00955

[6] L. Zhu , M. Zhang , J. Xu , C. Li , J. Yan , G. Zhou , W. Zhong , T. Hao , J. Song , X. Xue , Z. Zhou , R. Zeng , H. Zhu , C.‐C. Chen , R. C. I. Mackenzie , Y. Zou , J. Nelson , Y. Zhang , Y. Sun , F. Liu , Nat. Mater. 2022, 21, 656. 35513501 10.1038/s41563-022-01244-y

[7] Y. Cui , Y. Xu , H. Yao , P. Bi , L. Hong , J. Zhang , Y. Zu , T. Zhang , J. Qin , J. Ren , Z. Chen , C. He , X. Hao , Z. Wei , J. Hou , Adv. Mater. 2021, 33, 2102420. 10.1002/adma.202102420 34464466

[8] R. Sun , Y. Wu , X. Yang , Y. Gao , Z. Chen , K. Li , J. Qiao , T. Wang , J. Guo , C. Liu , X. Hao , H. Zhu , J. Min , Adv. Mater. 2022, 34, 2110147. 10.1002/adma.202110147 35438225

[9] Y. Cui , P. Zhu , H. Hu , X. Xia , X. Lu , S. Yu , H. Tempeld , R.‐A. Eichel , X. Liao , Y. Chen , Angew. Chem., Int. Ed. 2023, 62, e202304931. 10.1002/anie.202304931 37431837

[10] Z. Guo , A. K. Jena , G. M. Kim , T. Miyasaka , Energy Environ. Sci. 2022, 15, 3171.

[11] H. Lai , M. Guo , Y. Zhu , L. Chen , P. Tan , C. Yang , F. He , Mater. Chem. Front. 2021, 5, 1486.

[12] L. Yuan , S. Liang , C. Xiao , Q. Chen , W. Li , Chem. – Asian J. 2021, 16, 4171. 34738329 10.1002/asia.202101147

[13] S. Pang , X. Zhou , S. Zhang , H. Tang , S. Dhakal , X. Gu , C. Duan , F. Huang , Y. Cao , ACS Appl. Mater. Interfaces 2020, 12, 16531. 32192336 10.1021/acsami.0c01850

[14] J. Li , J. Song , H. Chen , Q. Wei , J. Yuan , H. Peng , Y. Zou , Sol. Energy 2021, 223, 100.

[15] Y. Chen , F. Bai , Z. Peng , L. Zhu , J. Zhang , X. Zou , Y. Qin , H. K. Kim , J. Yuan , L.‐K. Ma , J. Zhang , H. Yu , P. C. Y. Chow , F. Huang , Y. Zou , H. Ade , F. Liu , H. Yan , Adv. Energy Mater. 2020, 11, 2003141

[16] W. Gao , X. Ma , Q. An , J. Gao , C. Zhong , F. Zhang , C. Yang , J. Mater. Chem. A 2020, 8, 14583.

[17] G. Zhang , X.‐K. Chen , J. Xiao , P. C. Y. Chow , M. Ren , G. Kupgan , X. Jiao , C. C. S. Chan , X. Du , R. Xia , Z. Chen , J. Yuan , Y. Zhang , S. Zhang , Y. Liu , Y. Zou , H. Yan , K. S. Wong , V. Coropceanu , N. Li , C. J. Brabec , J.‐L. Bredas , H.‐L. Yip , Y. Cao , Nat. Commun. 2020, 11, 3943. 32770068 10.1038/s41467-020-17867-1 PMC7414148

[18] C. Li , J. Zhou , J. Song , J. Xu , H. Zhang , X. Zhang , J. Guo , L. Zhu , D. Wei , G. Han , J. Min , Y. Zhang , Z. Xie , Y. Yi , H. Yan , F. Gao , F. Liu , Y. Sun , Nat. Energy 2021, 6, 605.

[19] Y. Shi , Y. Chang , K. Lu , Z. Chen , J. Zhang , Y. Yan , D. Qiu , Y. Liu , M. A. Adil , W. Ma , X. Hao , L. Zhu , Z. Wei , Nat. Commun. 2022, 13, 3256. 35672325 10.1038/s41467-022-30927-y PMC9174259

[20] C. Zhu , J. Yuan , F. Cai , L. Meng , H. Zhang , H. Chen , J. Li , B. Qiu , H. Peng , S. Chen , Y. Hu , C. Yang , F. Gao , Y. Zou , Y. Li , Energy Environ. Sci. 2020, 13, 2459.

[21] M. Deng , H. Meng , X. Xu , J. Tang , L. Yu , R. Li , Q. Peng , Chem. Eng. J. 2022, 440, 135975.

[22] F. Cheng , Y. Cui , F. Ding , Z. Chen , Q. Xie , X. Xia , P. Zhu , X. Lu , H. Zhu , X. Liao , Y. Chen , Adv. Mater. 2023, 35, 2300400. 10.1002/adma.202300820 37073407

[23] P. Hohenberg , W. Kohn , Phys. Rev. 1964, 136, B864.

[24] W. Kohn , L. J. Sham , Phys. Rev. 1965, 140, A1133.

[25] Y. Cui , P. Zhu , X. Liao , Y. Chen , J. Mater. Chem. C 2020, 8, 15920.

[26] M. Mesta , J. H. Chang , S. Shil , K. S. Thygesen , J. M. G. Lastra , J. Phys. Chem. A 2019, 123, 4980. 31117588 10.1021/acs.jpca.9b02391

[27] P. Verma , D. G. Truhlar , Trends Chem. 2020, 2, 302.

[28] Z. Yao , X. Liao , K. Gao , F. Lin , X. Xu , X. Shi , L. Zuo , F. Liu , Y. Chen , A. K.‐Y. Jen , J. Am. Chem. Soc. 2018, 140, 2054. 29377679 10.1021/jacs.7b13239

[29] S. Li , L. Ye , W. Zhao , S. Zhang , S. Mukherjee , H. Ade , J. Hou , Adv. Mater. 2016, 28, 9423. 27606970 10.1002/adma.201602776

[30] L. Leonat , G. Sbarcea , I. Branzoi , UPB scientific Bulletin, Series B: Chemistry and Materials Science 2013, 75, 111.

[31] Y. Wu , J. Guo , R. Sun , J. Min , NPJ Computat. Mater. 2020, 6, 120.

[32] A. Mahmood , J.‐L. Wang , Energy Environ. Sci. 2021, 14, 90.

[33] G. J. Moore , O. Bardagot , N. Banerji , Adv. Theory Simulat. 2022, 5, 2100511CODEN.

[34] M. R. S. A. Janjua , A. Irfan , M. Hussien , M. Ali , M. Saqib , M. Sulaman , Energy Technol. 2022, 10, 2200019.

[35] Q. Zhang , Y. J. Zheng , W. Sun , Z. Ou , O. Odunmbaku , M. Li , S. Chen , Y. Zhou , J. Li , B. Qin , K. Sun , Adv. Sci. 2022, 9, e2104742. 10.1002/advs.202104742 PMC8867193 34989179

[36] W. Liu , W. Li , J. Yao , C. Zhan , Chin. Chem. Lett. 2018, 29, 381.

[37] X. Shi , L. Zuo , S. B. Jo , K. Gao , F. Lin , F. Liu , A. K.‐Y. Jen , Chem. Mater. 2017, 29, 8369.

[38] J. Wang , H. Yao , Y. Xu , L. Ma , J. Hou , Mater. Chem. Front. 2021, 5, 709.

[39] R. P. M. Iczkowski , J. L. Margrave , J. Am. Chem. Soc. 1961, 83, 3547.

[40] G. Cai , Y. Li , J. Wang , Y. Zhang , X. Lu , J. Lian , P. Zeng , Y. Wang , X. Zhan , ACS Appl. Polymer Mater. 2020, 3, 23.

[41] P. Bi , S. Zhang , J. Ren , Z. Chen , Z. Zheng , Y. Cui , J. Wang , S. Wang , T. Zhang , J. Li , Y. Xu , J. Qin , C. An , W. Ma , X. Hao , J. Hou , Adv. Mater. 2021, 34, 2108090. 10.1002/adma.202108090 34784077

[42] J. Gao , Y. Li , S. Li , X. Xia , X. Lu , M. Shi , H. Chen , Sol. Energy Mater. Sol. Cells 2021, 225, 111046.

[43] R. Hou , M. Li , X. Ma , H. Huang , H. Lu , Q. Jia , Y. Liu , X. Xu , H.‐B. Li , Z. Bo , ACS Appl. Mater. Interfaces 2020, 12, 46220. 32938186 10.1021/acsami.0c13993

[44] S. Li , L. Zhan , W. Zhao , S. Zhang , B. Ali , Z. Fu , T.‐K. Lau , X. Lu , M. Shi , C.‐Z. Li , J. Hou , H. Chen , J. Mater. Chem. A 2018, 6, 12132.

[45] D. Liu , L. Yang , Y. Wu , X. Wang , Y. Zeng , G. Han , H. Yao , S. Li , S. Zhang , Y. Zhang , Y. Yi , C. He , W. Ma , J. Hou , Chem. Mater. 2018, 30, 619.

