# Supplementary material for: Data Cleansing and Sub‐Unit‐Based Molecular Description Enable Accurate Prediction of The Energy Levels of Non‐Fullerene Acceptors Used in Organic Solar Cells
Source: Adv Sci (Weinh). 2024 Feb 22;11(17):2308652. doi: 10.1002/advs.202308652 (PMC11077656; doi:10.1002/advs.202308652)
Supplement: Supplementary file 1 — Supporting Information [file ADVS-11-2308652-s001.pdf]

ADVANCED SCIENCE
Open Access

Supporting Information

for *Adv. Sci.*, DOI 10.1002/advs.202308652

Data Cleansing and Sub-Unit-Based Molecular Description Enable Accurate Prediction of The Energy Levels of Non-Fullerene Acceptors Used in Organic Solar Cells

Ting Zhang, Joshua Yuk Lin Lai, Mingzhe Shi, Qing Li, Chen Zhang and He Yan**

Supporting Information

Data cleansing and sub-unit-based molecular description enable accurate prediction of the energy levels of non-fullerene acceptors used in organic solar cells

This supporting information presents the following contents.

Figure S1. Chemical structure of a representative non-fused NFA molecule.

Table S1. Examples of differences between DFT calculated and CV measured energy levels.

Table S2. Variation of CV measured energy level between different research groups for the same material.

Table S3. Variation of Voc and bandgap of Y6 measured between different research groups for the same active layer composition.

Table S4. Computed HOMO/LUMO energy values for common aromatic building blocks used in non-fullerene acceptors of organic solar cells.

Table S5. Computed HOMO/LUMO energy values for common end groups used in non-fullerene acceptors of organic solar cells.

Table S6. Common side chain substitution groups are used in non-fullerene acceptors of organic solar cells.

Table S7. Summary of the data for 20 “user-case” NFA molecules.

References

Figure S1. Chemical structure of a representative non-fused NFA molecule.

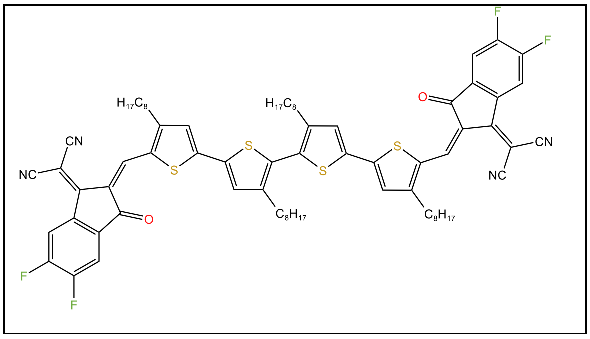

Table S1. Examples of differences between DFT calculated and CV measured energy levels.

Material	$LUMO_{(DFT)}$	$LUMO_{(CV)}$	ΔEv(LUMO)	$HOMO_{(DFT)}$	$HOMO_{(CV)}$	ΔEv(HOMO)
ITIC[1]	-3.41	-4.02	0.61	-5.55	-5.61	0.06
IT- M[1]	-3.35	-3.98	0.63	-5.5	-5.58	0.08
IT-DM[1]	-3.29	-3.93	0.64	-5.45	-5.56	0.11
Y6[2]	-3.52	-4.07	0.55	-5.55	-5.74	0.19
L8-BO[2]	-3.49	-3.9	0.41	-5.53	-5.68	0.15
L8-OD[2]	-3.49	-3.91	0.42	-5.55	-5.71	0.16
L8-HD[2]	-3.5	-3.9	0.4	-5.54	-5.71	0.17
DTPC-IC[3]	-3.29	-3.97	0.68	-5.03	-5.21	0.18
DTPC-DFIC[3]	-3.44	-4.1	0.66	-5.14	-5.31	0.17
		average	0.56		average	0.14

Table S2. Variation of CV measured energy level between different research groups for the same material.

Material/Y6	Ref. [2]	Ref. [4]	Ref. [5]	Ref. [6]	Max - Min	SD
$LUMO_{(CV)}$	-4.07	-4.04	-4.08	-3.89	0.19	0.088
$HOMO_{(CV)}$	-5.74	-5.56	-5.70	-5.71	0.18	0.080
Material/PM6	Ref. [7]	Ref. [2]	Ref. [8]	Ref. [9]	Max - Min	SD
$LUMO_{(CV)}$	-3.64	-3.47	-3.68	-3.60	0.21	0.091
$HOMO_{(CV)}$	-5.74	-5.56	-5.70	-5.71	0.18	0.080

Table S3. Variation of Voc and bandgap of Y6 measured between different research groups for the same active layer composition.

Active Layer/ PM6:Y6	Ref. [2]	Ref. [4]	Ref. [5]	Ref. [6]	Ref. [7]	Ref. [8]	Ref. [9]	Max - Min	SD
Voc	0.84	0.82	0.86	0.82	0.84	0.85	0.82	0.04	0.015
Y6 Bandgap	1.33	1.35	1.33	1.33	1.33	1.32	1.35	0.03	0.010

Table S4. Computed HOMO/LUMO energy values for common aromatic building blocks used in non-fullerene acceptors of organic solar cells.

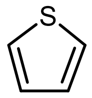	**LUMO: -0.23**<br>**HOMO: -6.35**	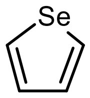	**LUMO: -0.35**<br>**HOMO: -6.32**
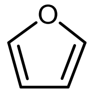	**LUMO: 0.51**<br>**HOMO: -6.12**	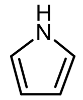	**LUMO: -3.68**<br>**HOMO: -6.78**
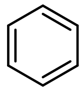	**LUMO: 0.07**<br>**HOMO: -6.72**	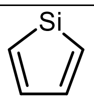	**LUMO: -3.27**<br>**HOMO: -5.90**
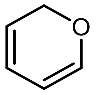	**LUMO: -0.38**<br>**HOMO: -5.14**	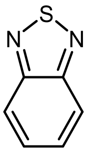	**LUMO: -2.35**<br>**HOMO: -6.62**
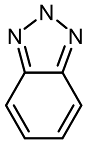	**LUMO: -1.88**<br>**HOMO: -7.34**	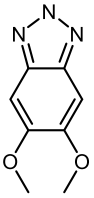	**LUMO: -1.33**<br>**HOMO: -6.06**
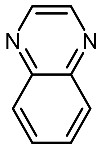	**LUMO: -1.93**<br>**HOMO: -6.70**	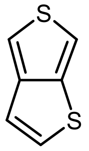	**LUMO: -0.90**<br>**HOMO: -5.57**
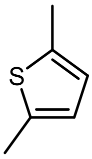	**LUMO: 3.90**<br>**HOMO: -8.32**	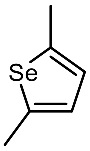	**LUMO: -0.16**<br>**HOMO: -5.76**
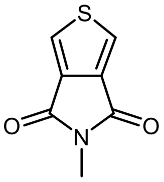	**LUMO: -2.08**<br>**HOMO: -7.09**	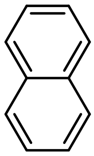	**LUMO: -0.98**<br>**HOMO: -5.80**

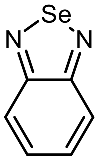	**LUMO: -2.44**<br>**HOMO: -6.47**	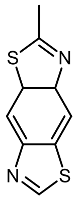	**LUMO: -1.81**<br>**HOMO: -5.66**
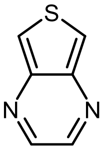	**LUMO: -2.28**<br>**HOMO: -6.24**	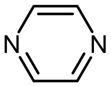	**LUMO: -1.43**<br>**HOMO: -6.80**
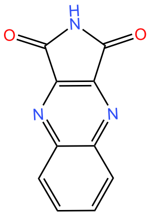	**LUMO: -2.75**<br>**HOMO: -7.12**	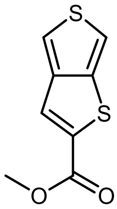	**LUMO: -1.80**<br>**HOMO: -5.80**
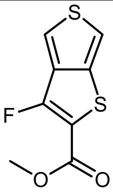	**LUMO: -1.87**<br>**HOMO: -5.89**		

Table S5. Computed HOMO/LUMO energy values for common end groups used in non-fullerene acceptors of organic solar cells.

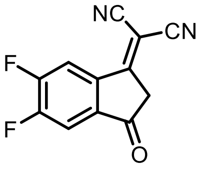	**IC-2F**<br>**LUMO: -3.40**	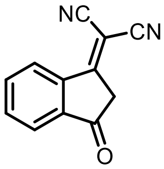	**IC**<br>**LUMO: -3.19**
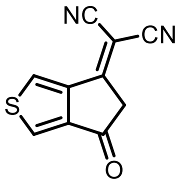	**IC-Th-1**<br>**LUMO: -3.21**	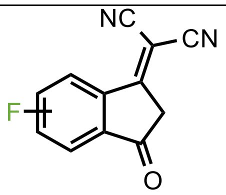	**IC-F-1**<br>**LUMO: -3.33**
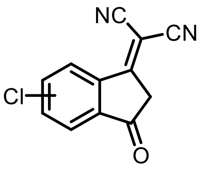	**IC-Cl-1**<br>**LUMO: -3.38**	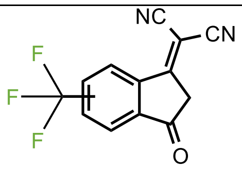	**IC-Tf**<br>**LUMO: -3.50**
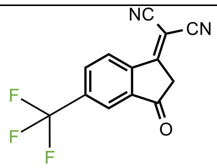	**IC-F3**<br>**LUMO: -3.61**	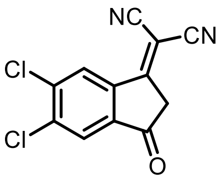	**IC-2Cl**<br>**LUMO: -3.50**

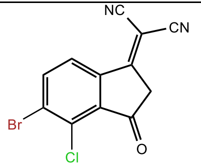	**IC-ClBr-2**<br>**LUMO: -3.47**	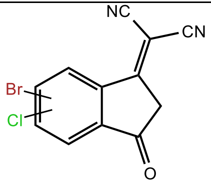	**IC-ClBr-1**<br>**LUMO: -3.48**
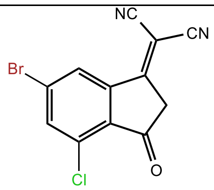	**IC-ClBr-3**<br>**LUMO: -3.52**	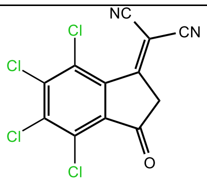	**IC-4Cl**<br>**LUMO: -3.71**
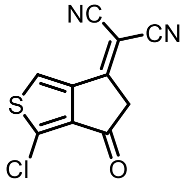	**IC-ThCl-1**<br>**LUMO: -3.22**	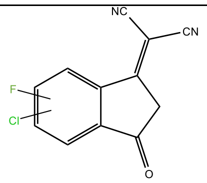	**IC-FCl**<br>**LUMO: -3.42**
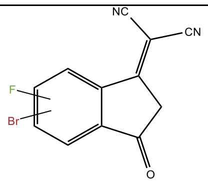	**IC-FBr**<br>**LUMO: -3.39**	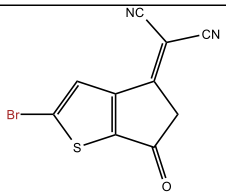	**IC-ThBr-2**<br>**LUMO: -3.46**
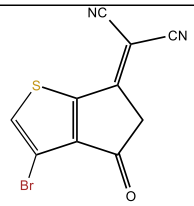	**IC-ThBr-3**<br>**LUMO: -3.55**	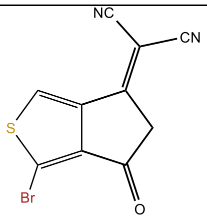	**IC-ThBr-1**<br>**LUMO: -3.19**
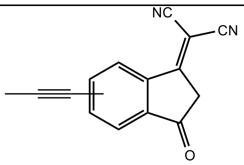	**IC-yle-1**<br>**LUMO: -3.13**	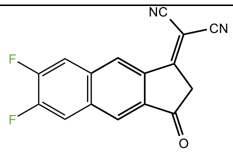	**IC-2FNp-2**<br>**LUMO: -3.25**
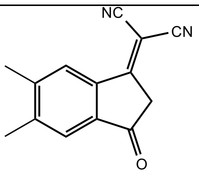	**IC-2Me**<br>**LUMO: -3.02**	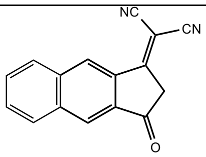	**IC-Np**<br>**LUMO: -3.09**
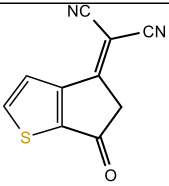	**IC-Th**<br>**LUMO: -3.20**	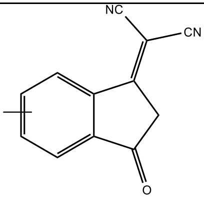	**IC-Me**<br>**LUMO: -3.18**
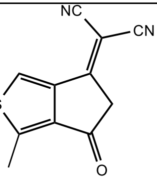	**IC-ThMe**<br>**LUMO: -2.99**	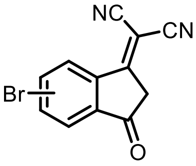	**IC-Br**<br>**LUMO: -3.37**

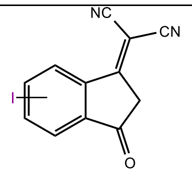	**IC-I**<br>**LUMO: -1.89**	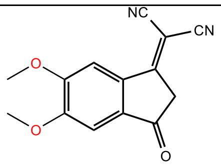	**IC-2MeO**<br>**LUMO: -2.94**
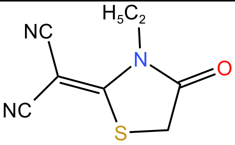	**Rh-1**<br>**LUMO: -2.84**	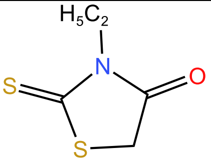	**Rh-7**<br>**LUMO: -1.93**
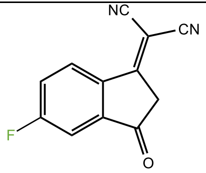	**IC-F-2**<br>**LUMO: -3.33**	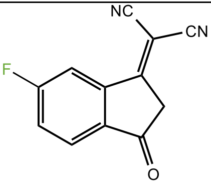	**IC-F-3**<br>**LUMO: -3.33**
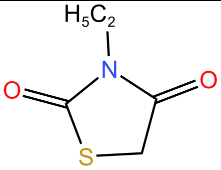	**Rh-8**<br>**LUMO: -0.92**	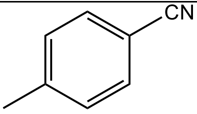	**Ph-CN**<br>**LUMO: -1.17**
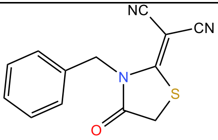	**Rh-5**<br>**LUMO: -2.31**	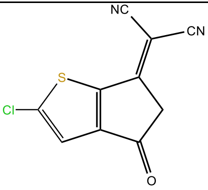	**IC-ThCl-3**<br>**LUMO: -3.55**
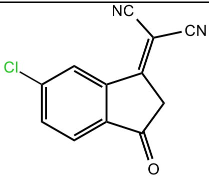	**IC-Cl-1**<br>**LUMO: -3.39**	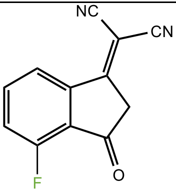	**IC-F-4**<br>**LUMO: -3.50**
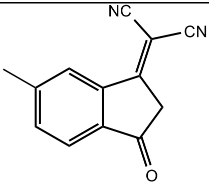	**IC-Me**<br>**LUMO: -3.27**	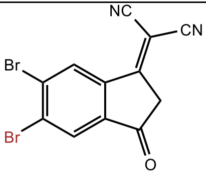	**IC-2Br**<br>**LUMO: -3.66**
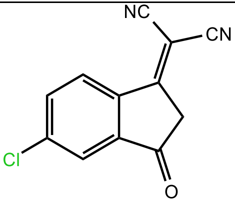	**IC-Cl-2**<br>**LUMO: -3.36**	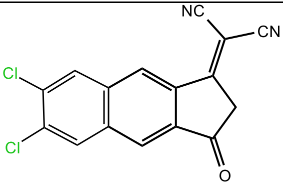	**IC-Ph-2Cl**<br>**LUMO: -3.36**
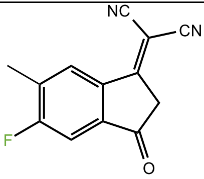	**IC-MeF**<br>**LUMO: -3.32**	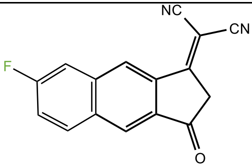	**IC-NF-1**<br>**LUMO: -3.30**

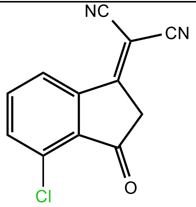	**IC-Cl-3**<br>**LUMO: -3.54**	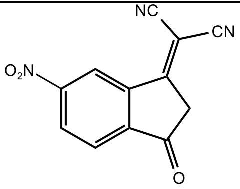	**IC-NO2-1**<br>**LUMO: -4.50**
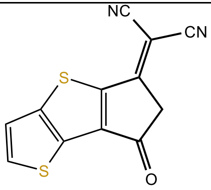	**IC-TT-1**<br>**LUMO: -3.23**	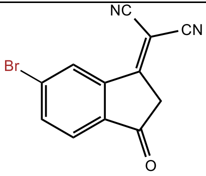	**IC-Br-1**<br>**LUMO: -3.37**
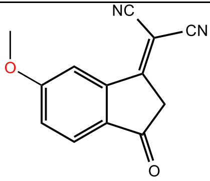	**IC-MeO-1**<br>**LUMO: -3.30**	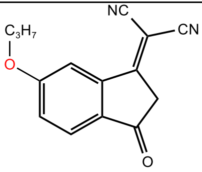	**IC-MeO-C3H7**<br>**LUMO: -3.27**
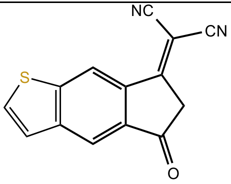	**IC-Ph-T**<br>**LUMO: -3.03**	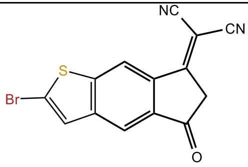	**IC-TBr**<br>**LUMO: 0**
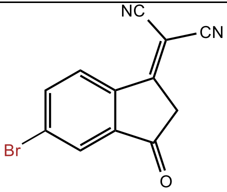	**IC-Br-2**<br>**LUMO: -3.36**	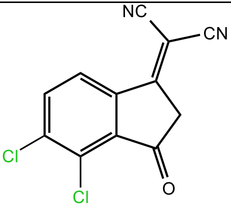	**IC-2Cl-1**<br>**LUMO: -3.68**
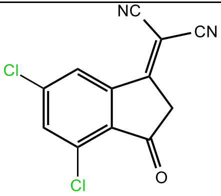	**IC-2Cl-2**<br>**LUMO: -3.72**	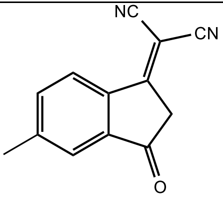	**IC-Ph-Me-4**<br>**LUMO: -3.25**
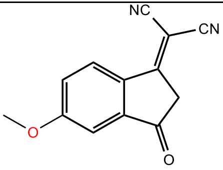	**IC-MeO-4**<br>**LUMO: -3.13**	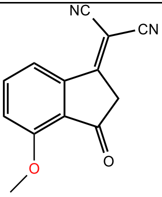	**IC-MeO-5**<br>**LUMO: -3.24**
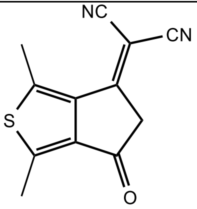	**IC-T2Me**<br>**LUMO: -2.97**	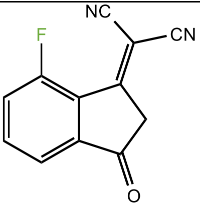	**IC-F-5**<br>**LUMO: -3.42**

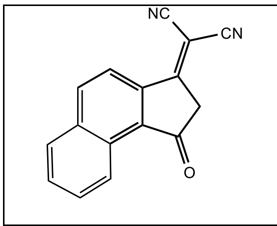	**IC-Ph**<br>**LUMO: -3.44**	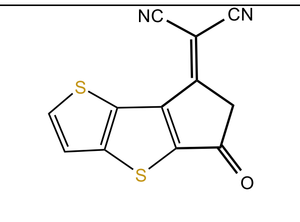	**IC-TT-2**<br>**LUMO: -3.23**
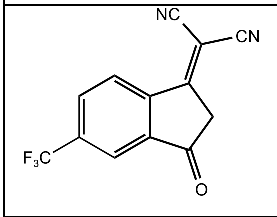	**IC-F3-2**<br>**LUMO: -3.61**	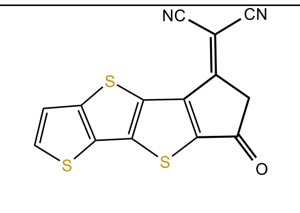	**IC-TTT**<br>**LUMO: -3.15**
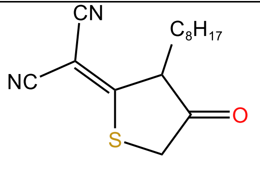	**Rh-3**<br>**LUMO: -2.28**	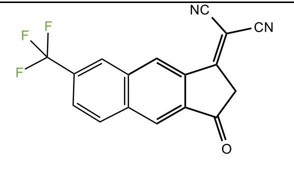	**IC-NF3**<br>**LUMO: -3.40**
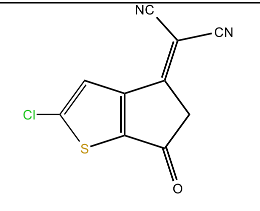	**IC-ThCl-2**<br>**LUMO: -3.49**	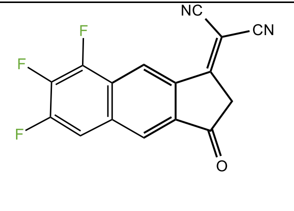	**IC-N3F**<br>**LUMO: -3.42**
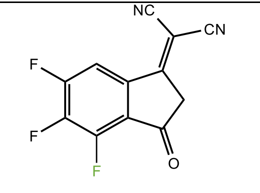	**IC-3F**<br>**LUMO: -3.70**		

Table S6. Common side chain substitution groups are used in non-fullerene acceptors of organic solar cells.

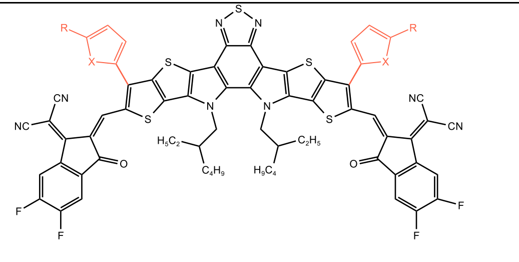<br>**+0.1**	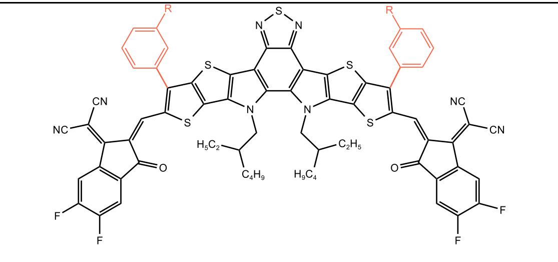<br>**+0.1**
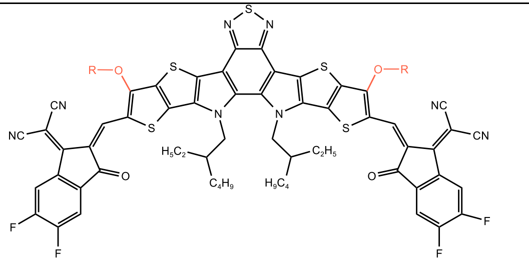<br>**+0.3**	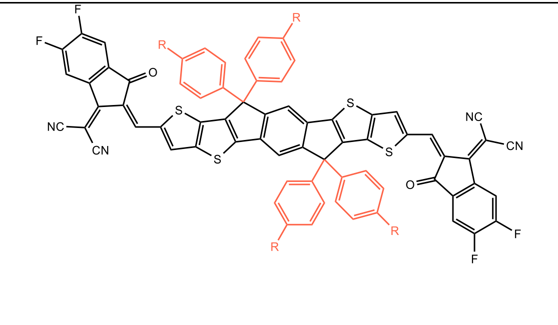

	**+0.1**
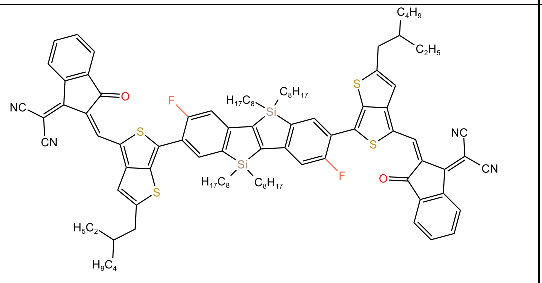 **-0.1**	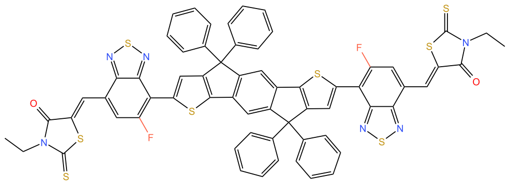 **-0.1**
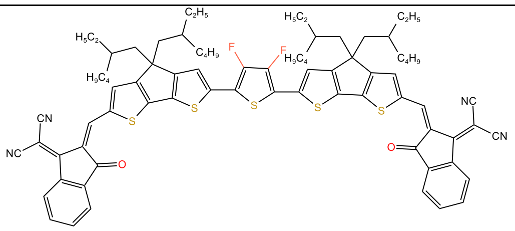 **-0.2**	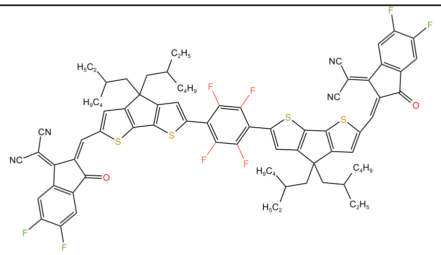 **-0.1**

Table S7. Summary of the data for 20 "user-case" NFA molecules.

#	SMA name	HOMO reported	LUMO reported	HOMO DFT	LUMO DFT	HOMO predict	LUMO predict	Donor polymer	HOMO Donor	Voc Predict	Voc report	delta Voc
1	BTP-eC9	-5.64	-4.05	-5.65	-3.63	-5.52	-4.06	PBDB-TF	-5.49	0.83	0.84	0.01
2	BTP-Th	-5.75	-3.96	-5.53	-3.47	-5.70	-4.00	PTQ10	-5.54	0.94	0.87	0.07
3	COi8DFIC	-5.50	-3.88	-5.66	-3.79	-5.50	-3.98	PTB7-Th	-5.22	0.64	0.68	0.04
4	EH-IDTBR	-5.58	-3.90	-5.38	-3.53	-5.47	-3.68	P3HT	-5.01	0.73	0.76	0.03
5	FBR	-5.83	-3.75	-5.88	-3.44	-5.60	-3.71	PffBT4T-2DT	-5.34	1.03	1.12	0.09
7	IDFBR	-5.75	-3.70	-5.73	-3.39	-5.49	-3.66	PffBT4T-2DT	-5.34	1.08	1.08	0.00
8	IDIC	-5.69	-3.91	-5.95	-3.69	-5.65	-3.93	PTQ10	-5.54	1.01	0.962	0.05
9	IDT-2BR	-5.52	-3.69	-5.4	-3.52	-5.44	-3.80	P3HT	-5.01	0.61	0.84	0.23
10	IEIC	-5.48	-3.85	-5.25	-3.29	-5.44	-3.79	PTB7-Th	-5.22	0.83	0.95	0.12
11	IEICO	-5.31	-3.91	-5.06	-3.3	-5.34	-3.72	J52	-5.19	0.87	0.87	0.00
12	IHIC	-5.45	-3.93	-5.66	-3.7	-5.45	-3.95	PTB7-Th	-5.22	0.67	0.754	0.09
13	INIC	-5.45	-3.88	-5.63	-3.55	-5.56	-3.82	FTAZ	-5.41	0.99	0.957	0.04
14	IT-4F	-5.66	-4.14	-5.59	-3.5	-5.61	-3.98	PBDB-T-SF	-5.38	0.80	0.88	0.08
15	IT-DM	-5.56	-3.93	-5.45	-3.29	-5.47	-3.77	PBDB-T	-5.31	0.94	0.97	0.03
16	ITIC	-5.50	-3.89	-5.48	-3.36	-5.59	-3.89	PBDB-T	-5.31	0.82	0.93	0.11
17	ITIC-Th	-5.66	-3.93	-5.61	-3.51	-5.59	-3.89	PTB7-Th	-5.22	0.73	0.8	0.07
18	IT-M	-5.58	-3.98	-5.5	-3.35	-5.59	-3.89	PBDB-T	-5.31	0.82	0.94	0.12
19	L8-BO	-5.68	-3.90	-5.53	-3.49	-5.53	-4.04	PBDB-TF	-5.49	0.85	0.87	0.02
20	Y6	-5.65	-4.10	-5.62	-3.58	-5.53	-4.04	PBDB-TF	-5.49	0.85	0.86	0.01
											Average of delta Voc	0.06

Reference

(1) Li, S.; Ye, L.; Zhao, W.; Zhang, S.; Mukherjee, S.; Ade, H.; Hou, J. Energy-Level Modulation of Small-Molecule Electron Acceptors to Achieve over 12% Efficiency in Polymer Solar Cells. *Adv. Mater.* **2016**, *28* (42), 9423–9429.

(2) Li, C.; Zhou, J.; Song, J.; Xu, J.; Zhang, H.; Zhang, X.; Guo, J.; Zhu, L.; Wei, D.; Han, G.; Min, J.; Zhang, Y.; Xie, Z.; Yi, Y.; Yan, H.; Gao, F.; Liu, F.; Sun, Y. Non-Fullerene Acceptors with Branched Side Chains and Improved Molecular Packing to Exceed 18% Efficiency in Organic Solar Cells. *Nat. Energy 2021 66* **2021**, *6* (6), 605–613.

(3) Yao, Z.; Liao, X.; Gao, K.; Lin, F.; Xu, X.; Shi, X.; Zuo, L.; Liu, F.; Chen, Y.; Jen, A. K. Y. Dithienopicenocarbazole-Based Acceptors for Efficient Organic Solar Cells with Optoelectronic Response over 1000 Nm and an Extremely Low Energy Loss. *J. Am. Chem. Soc.* **2018**, *140* (6), 2054–2057.

(4) Zhang, G.; Chen, X. K.; Xiao, J.; Chow, P. C. Y.; Ren, M.; Kupgan, G.; Jiao, X.; Chan, C. C. S.; Du, X.; Xia, R.; Chen, Z.; Yuan, J.; Zhang, Y.; Zhang, S.; Liu, Y.; Zou, Y.; Yan, H.; Wong, K. S.; Coropceanu, V.; Li, N.; Brabec, C. J.; Bredas, J. L.; Yip, H. L.; Cao, Y. Delocalization of Exciton and Electron Wavefunction in Non-Fullerene Acceptor Molecules Enables Efficient Organic Solar Cells. *Nat. Commun. 2020 111* **2020**, *11* (1), 1–10.

(5) Deng, M.; Meng, H.; Xu, X.; Tang, J.; Yu, L.; Li, R.; Peng, Q. Unique W-Shape Y6 Isomer as Effective Solid Additive for High-Performance PM6:Y6 Polymer Solar Cells. *Chem. Eng. J.* **2022**, *440*, 135975.

(6) Chen, Y.; Bai, F.; Peng, Z.; Zhu, L.; Zhang, J.; Zou, X.; Qin, Y.; Kyung Kim, H.; Yuan, J.; Ma, L.-K.; Zhang, J.; Yu, H.; Y Chow, P. C.; Huang, F.; Zou, Y.; Ade, H.; Liu, F.; Yan, H.; Chen, Y.; Bai, F.; Zhang, J.; Zou, X.; Kim, H. K.; Ma, L.; Yu, H.; Y Chow, P. C.; Yan Hong Kong, H.; Yan, H.; Peng, Z.; Qin, Y.; Ade, H.; Zhu, L.; Liu, F.; Yuan, J.; Zou, Y.; Huang, F. Asymmetric Alkoxy and Alkyl Substitution on Nonfullerene Acceptors Enabling High-Performance Organic Solar Cells. *Adv. Energy Mater.* **2021**, *11* (3), 2003141.

(7) Gao, W.; Ma, X.; An, Q.; Gao, J.; Zhong, C.; Zhang, F.; Yang, C. An Asymmetrical Fused-Ring Electron Acceptor Designed by a Cross-Conceptual Strategy Achieving 15.6%

Efficiency. *J. Mater. Chem. A* **2020**, *8* (29), 14583–14591.

(8) Shi, Y.; Chang, Y.; Lu, K.; Chen, Z.; Zhang, J.; Yan, Y.; Qiu, D.; Liu, Y.; Adil, M. A.; Ma, W.; Hao, X.; Zhu, L.; Wei, Z. Small Reorganization Energy Acceptors Enable Low Energy Losses in Non-Fullerene Organic Solar Cells. *Nat. Commun. 2022 131* **2022**, *13* (1), 1–10.

(9) Zhu, C.; Yuan, J.; Cai, F.; Meng, L.; Zhang, H.; Chen, H.; Li, J.; Qiu, B.; Peng, H.; Chen, S.; Hu, Y.; Yang, C.; Gao, F.; Zou, Y.; Li, Y. Tuning the Electron-Deficient Core of a Non-Fullerene Acceptor to Achieve over 17% Efficiency in a Single-Junction Organic Solar Cell. *Energy Environ. Sci.* **2020**, *13* (8), 2459–2466.
